# How I Met Your V2X Sensor Data: Analysis of Projection-Based Light Field Visualization for Vehicle-to-Everything Communication Protocols and Use Cases

**DOI:** 10.3390/s23031284

**Published:** 2023-01-22

**Authors:** Peter A. Kara, Andras Wippelhauser, Tibor Balogh, Laszlo Bokor

**Affiliations:** 1 Department of Networked Systems and Services, Faculty of Electrical Engineering and Informatics, Budapest University of Technology and Economics, Műegyetem rkp. 3., H-1111 Budapest, Hungary; 2 Wireless Multimedia and Networking Research Group, Department of Computer Science, Faculty of Science, School of Computer Science and Mathematics, Engineering and Computing, Kingston University, Penrhyn Road Campus, Kingston upon Thames, London KT1 2EE, UK; 3 Holografika, 1192 Budapest, Hungary

**Keywords:** light field, projection-based light field visualization, V2X-based environment sensing, visualization of sensor data, cooperative intelligent transport systems, V2X communication protocols

## Abstract

The practical usage of V2X communication protocols started emerging in recent years. Data built on sensor information are displayed via onboard units and smart devices. However, perceptually obtaining such data may be counterproductive in terms of visual attention, particularly in the case of safety-related applications. Using the windshield as a display may solve this issue, but switching between 2D information and the 3D reality of traffic may introduce issues of its own. To overcome such difficulties, automotive light field visualization is introduced. In this paper, we investigate the visualization of V2X communication protocols and use cases via projection-based light field technology. Our work is motivated by the abundance of V2X sensor data, the low latency of V2X data transfer, the availability of automotive light field prototypes, the prevalent dominance of non-autonomous and non-remote driving, and the lack of V2X-based light field solutions. As our primary contributions, we provide a comprehensive technological review of light field and V2X communication, a set of recommendations for design and implementation, an extensive discussion and implication analysis, the exploration of utilization based on standardized protocols, and use-case-specific considerations.

## 1. Introduction

Hungarian inventor and architect Ernő Rubik once said that “the Cube is, at the same time, a symbol of simplicity and complexity”. In a sense, we can look at our personal automobiles of the current era in a very similar fashion. On the one hand, there is a definite simplicity of driving due to the many supportive and automated functionalities of modern vehicles. On the other hand, this ease of usage is enabled by the simple fact that cars are more complex than ever before. This complexity partially lies in the sheer amount of sensors contained by a single vehicle. One could say that cars are slowly but surely becoming sensors on wheels.

Vehicles are not the only entities in the entirety of Intelligent Transport Systems (ITS) that carry sensors. The vicinity of the road infrastructure is typically swarmed by different sensor technologies. From the pressure-sensitive and electromagnetic devices built into the road itself to road-side equipment and traffic gates that use optical technologies, laser, and radar, the infrastructure of the modern traffic ecosystem is not only rich in diverse sensors but, similarly to vehicles, continues to gain more and more.

The information collected by the sensors of the infrastructure is communicated to the vehicles via several different use cases and protocols. We call this infrastructure-to-vehicle (I2V) communication. However, in the opposite direction, vehicle-to-infrastructure (V2I) communication is equally relevant, as the information collected by the sensors of the vehicle may also contribute a lot to the safety and efficiency of the traffic. This latter is not limited to data regarding the vehicle itself (e.g., current position, orientation, speed, acceleration, tire pressure, etc.) but extends to its surroundings as well. For instance, the cameras of vehicles may perceive traffic anomalies and incidents.

One way for such information to reach another vehicle is through a chain of V2I and I2V communications. However, vehicles may directly distribute information among each other through vehicle-to-vehicle (V2V) communication. Since vehicles may communicate with any appropriate entity in the modern traffic ecosystem, at the time of writing this paper, vehicle-to-everything or vehicle-to-anything (V2X) is already a widely-used terminology. As vehicles may cooperate via the sharing of sensor information, their collective perception of the traffic environment—commonly known as cooperative perception—and the general collaboration between vehicles usher in an age of cooperative ITS (C-ITS).

**Figure 1:**
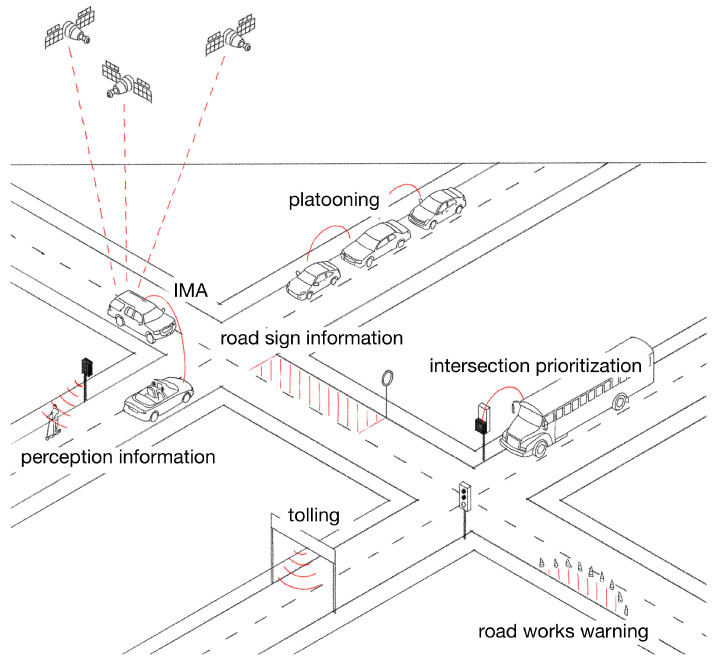
Intelligent transport systems: a generic view with example use cases.

Some example V2V, V2I, and I2V use cases are collected in Figure 1. In the top left corner of the figure, two vehicles are approaching an intersection at the same time so that they might collide. Via V2V messaging, the two vehicles are able to detect the possible collision, and they can perform an evasive maneuver to mitigate the risk. As seen in the top middle of the picture, the vehicles follow each other closely. They operate in a platooning mode, which is enabled by V2V communication. The vulnerable road users can also be equipped with an ITS station, or they can be detected by infrastructure sensors. In the middle of the picture, we can see that V2X enables the digitalization of road signs, which can increase awareness through I2V use cases. The right crossing is a signalized intersection with a prioritization function. The bus obtains priority over the other traffic participants thanks to the V2X communication. At the bottom of the figure, we can see a tolling gate that ensures safe, efficient, and fast fee collection through V2I use cases. A road work involving lane closure is represented on the bottom right of the picture. Such I2V use cases protect the road workers and the vehicles’ occupants too. The exact digital representation of the various road events helps the vehicle to mitigate potential upcoming risks.

At this point, one may easily think that all that was stated so far is constrained to autonomous, self-driving vehicles. While it is true that the vast majority of ITS communication happens seamlessly, one must note that a great number of use cases provide information to the driver of the vehicle. It is quite common that such information is displayed by the smartphone or other smart device of the driver, which, in fact, is rather suboptimal in terms of safety, as the visual attention of the driver should always be on the traffic [1]. This is also applicable to integrated onboard displays [2].

A relatively straightforward approach is to use the windshield as a modern translucent display to convey the information. In such a scenario, the issues related to visual attention are averted. However, if the implemented visualization solution was flat 2D, then the driver would need to endure the difficulties of switching and adjusting between the 2D contents and the 3D reality of traffic. The visualized contents in this context are not necessarily limited to textual data; information can be visualized as any graphical data, such as symbols, arrows (e.g., perceptually projected onto the topography of the road), map segments, and more.

A near-eye 3D technology that could be considered for such a usage context is augmented reality (AR). While it may be indeed beneficial for certain professional and warfare applications, its near-eye nature may not be adequate for the use case of driving. More specifically, adding a personal viewing gear to the context may introduce unnecessary difficulties and may potentially make it cumbersome. Technically speaking, the drawbacks may outweigh the benefits.

In order to utilize 3D visuals without the need for viewing gear, one must rely on autostereoscopic, glasses-free 3D visualization. Projection-based light field visualization satisfies such requirements, as it creates a field of light, the perception of which is analogous to our 3D reality. Although the emergence of this technology is still in its rather early phase, it may serve as an excellent candidate for information visualization in the automotive industry.

Using light field visualization has numerous benefits. First and foremost, it does not require any viewing gear whatsoever. Even without considering the tactile sensitivity of specific vulnerable users [3,4,5], the lack of the need for some sort of a viewing device is a convenience, or rather, the opposite can be a significant inconvenience. After all, in the most generic use case, the individual is expected to operate a personal vehicle and not a jet fighter. The autostereoscopic 3D visualization itself provides a realistic parallax effect for the driver. Depending on the implementation of the system, it may either be limited to the horizontal direction or it may support both directions. As the view of the real world and the utilization of V2X sensor data in synthesized visuals are combined in front of the driver, the light rays of such projection-based technology may produce a more seamless integration of the two. Trivially, as one of the fundamental goals of such solutions is that the driver may observe the traffic and the displayed information simultaneously (i.e., without the need to divert attention away from the traffic), the driver is thus expected to view the visualization for extended periods, if the travel demands it. At the time of writing this paper, no scientific work reports perceptual fatigue and simulator sickness associated with projection-based light field visualization, unlike other technologies [6,7,8,9,10,11].

In this paper, we address the utilization of projection-based light field technology to visualize V2X communication protocols. As the primary contribution of this work, we investigate both V2X and light field technologies, based on which a set of recommendations is provided. Additionally, we discuss considerations relevant to the design, implementation, and utilization, and elaborate on the associated implications.

According to the best knowledge of the authors, this is the first work to consider V2X sensor data in the context of automotive light field visualization. Related research efforts study the hardware-in-the-loop simulation of autonomous vehicles [12], light field imaging for autonomous underwater vehicles (AUVs) [13,14], zero-latency motion visualization [15], and AR [16,17,18,19,20] automotive head-up displays (HUDs). Light field HUDs and windshields (or windscreens) are addressed by several works [21,22,23,24,25,26]; however, none of them study V2X sensor data. For example, the recent work by Murugan et al. [27] focused solely on information about the autonomous vehicle in which the AR HUD was situated. Yöntem et al. [28] provided a comprehensive overview of such immersive interfaces and highlighted how warnings regarding road hazards could be aligned spatially to match hazard direction and location. The same applies to the recent review by Skirnewskaja and Wilkinson [29].

Regarding the scope of our work, the paper does not analyze specific sensor technologies (e.g., [30,31,32]). Instead, the emphasis is on utilizing the collected V2X sensor data. While vehicles are, in fact, both producers and receivers of sensor data in the V2X communication ecosystem, the focus of this paper is narrowed to the receiver side.

The main motivations, contributions, and benefits of this work are briefly summarized in the following lists.

Motivations:

— There is an abundance of V2X sensor data.
— Automotive technologies are advanced enough to benefit from V2X use cases.
— Non-autonomous driving and non-remote driving are still dominant.
— Light field visualization can already be adapted to in-vehicle usage contexts.
— There is a lack of V2X-based light field solutions.
— The latency of V2X data transfer is sufficiently low.

Contributions:

— Comprehensive technological review of light field and V2X communication.
— A set of recommendations for design and implementation.
— Extensive discussion and implication analysis.
— Exploration of utilization based on standardized protocols.
— Use-case-specific considerations.

Benefits:

— The driver does not need to divert attention from the traffic.
— Light field visualization does not require viewing devices.
— Light field technology enables direction-aware visualization.
— Low-latency warnings support traffic safety.
— V2X use cases support traffic efficiency in general.
— Visualization of direction–selective contents.
— Miscellaneous usage potentials for autonomous vehicles.

The remainder of this paper is structured as follows. Section 2 reviews the sensor data, protocols, and relevant use cases in the context of V2X communication. Section 3 elaborates on the technological background and the utilization considerations of projection-based light field technology. The paper provides a set of recommendations for V2X information visualization in Section 4. Section 5 further discusses the vital aspects and implications of projection-based 3D imaging systems in the investigated context. The paper is concluded in Section 6, which also summarizes the next steps toward such solutions.

## 2. V2X Protocols and Use Cases

### 2.1. General Overview of Operation

**Figure 2:**
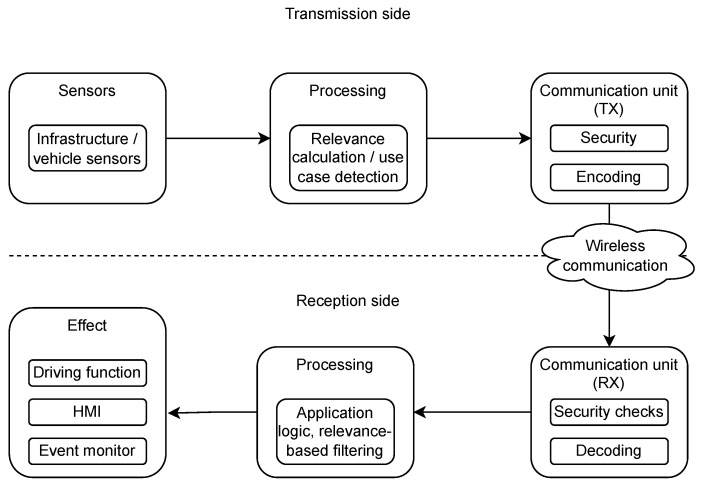
Generic V2X information pipeline.

In this section, we provide an abstract model of the V2X use cases, as shown in Figure 1, in order to enhance the understanding of complex V2X-based perception systems. V2X protocols implement a distributed communication scheme, meaning that the data provided by an ITS station is shared with all neighboring ITS stations. The communication typically does not have a session or a similar stateful connection so that the operation can be more or less accurately described via the life cycle of a single packet. The related pipeline is depicted in Figure 2.

**Figure 3:**
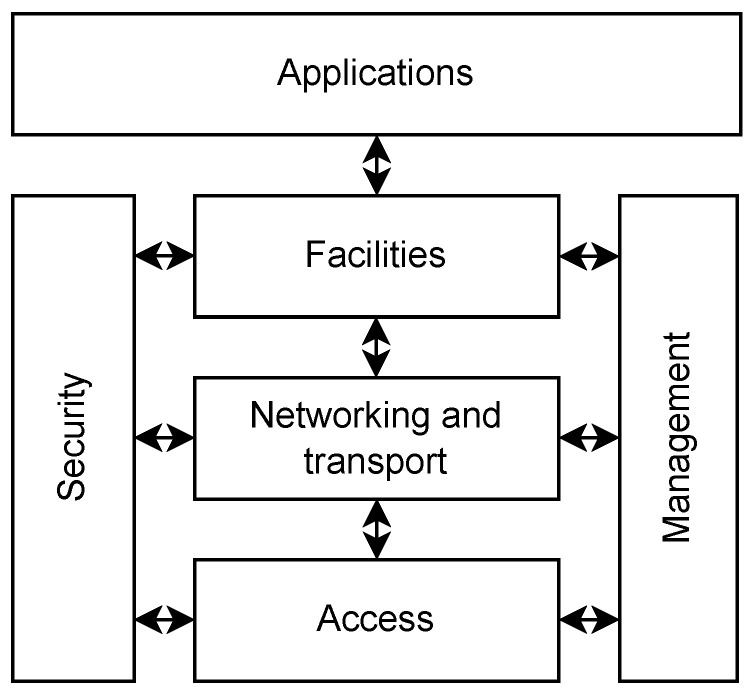
ITS four-layer architecture [33,34].

The actors in the V2X communication are so-called ITS stations. The communication scheme follows the ITS four-layer architecture [33,34], as depicted in Figure 3. The two major access layer technologies are 802.11p [35] and C-V2X PC5 [36]. In order to meet the requirements of new services, the successors of these standards are currently under development [37,38,39,40] (802.11bd and 5G NR, respectively). Network and transport layer services, such as GeoNet [41], BTP [42], and WAVE [43], are responsible for message transmission and discrimination. The facility layer services describe the message formats to be exchanged to support the various use cases. Such services are defined typically by ETSI [44,45,46,47,48,49] and SAE [50]. The application layer is responsible for message management and triggering conditions [51,52,53,54,55,56,57,58,59,60,61,62,63,64,65]. The management layer helps the cooperation of the other layers [66], while the security layer [67,68,69,70] lays the foundation to establish trust across the stations. This architecture is designed and tailored for V2X use cases, sharing relevant standardized information across the network. In the V2X system, sensors are installed to produce the necessary data to be shared over the V2X network. Sensors include infrastructure (such as loop detector, camera, lidar, radar, or manual road operator input) and vehicular detectors (such as GPS, IMU, radar, lidar, etc.). The subject of the detection can be the state of the ITS station or the state of the environment as well. The applicable sensors are discussed in the following sections.

The available sensor detections are filtered, validated, possibly fused, and processed. After that, the digested raw data can be used to generate and send V2X messages. The communication unit offers an API where the sensor detections can be provided to be shared as standardized messages in the V2X network. This API is present regardless of the used V2X facility service. Besides the details of the sensor information, the interface might include other metadata, such as the validity period of the detection.

The communication unit uses the preprocessed sensor detections received from the API to assemble standard V2X messages. It is essential to have a common understanding of the standard format of the message among the ITS stations. The communication unit then creates cryptographic signatures for the messages, ensuring integrity, authenticity, and non-repudiation. Then, the network layer of the message is assembled, and the packet is handed over to the radio layer. The radio layer queues the transmission request. When the medium access control (MAC) enables the transmission, the adequately encoded packet is sent to the air. The transmission mode in the C-ITS world is typically broadcast.

The receiver communication unit obtains the radio signals of the sender device. The physical layer decodes the message and forwards it to the upper layers. In the MAC layer, the communication unit tests whether it should receive a particular message. The network layer checks the packet relevance. Security checks are also performed in the network layer. The correctness of the signature is validated, and the presence and validity of the certificate chain are also examined. In the case of a successful validation, the received packet is considered trusted. After a simple API conversion logic, the communication unit passes the message to the reception-side processing modules.

The reception-side processing unit is responsible for processing and selecting the relevant information for the users. The reception side can be an infrastructure device or a vehicle as well. The operation of the processing unit highly depends on the supported and momentarily active applications. The applications can include data collection for the infrastructure operator, context-dependent warnings and awareness services for the human drivers, or sensor-type input for automated driving functions. In the scope of this article, the warnings and awareness services for human drivers are the most relevant use cases where the receiver-side ITS station is a vehicle. The processing unit filters the relevant road information, events, and moving objects based on the relevance defined by the active applications.

Eventually, the relevant data are forwarded to the Human-Machine Interface (HMI) by the processing unit. The HMI represents the essential features of the notification to the driver. It is crucial to design the HMI adequately from a user experience perspective. The driver’s attention should not be distracted by unwanted false positive notifications. Yet, the awareness of the potential danger needs to be raised in advance to save time for the driver for avoidance maneuvers. The timing of the notification is also essential; therefore, the requirements of a proper HMI solution in the vehicular context pose serious challenges to tackle. Olaverri-Monreal et al. [71] analyzed a wide variety of display layout designs and studied the drivers’ performance and gaze with the preferred locations for in-vehicle information presentation for Advanced Driver Assistance Systems (ADAS) and Driver Information Systems (DIS). A comprehensive summary and analysis of the previously published works in the field of HMI applications for use in a vehicular context are provided by Olaverri-Monreal and Jizba [72]. The authors studied human-machine interaction solutions, particularly those which address messages of cooperative ITS, providing a detailed background, a broad set of approaches, and example applications for the available concepts in the field. This work provides insight into options and best practices on how to implement safe HMI solutions with cooperative ADAS based on V2X. The authors also collect the complexities of the design and adaptation of in-vehicle systems that ensure road safety and emphasize that HMI systems based on V2X provide information way beyond the scope of today’s in-vehicle HMIs that do not have capabilities to support cooperative ITS services and applications. In line with this and to overcome limitations of existing HMI techniques, recent efforts started to investigate AR-based ADAS, which effectively visualizes information delivered through V2X communication in several evaluated scenarios, such as for pedestrian collision warning [73] and unsignalized intersection crossing [74].

Based on the literature, we conclude that novel placement options to display information conveyed by V2X technologies should be addressed, focusing on exploiting the possible benefits of advanced visualization paradigms, such as projection-based light field visualization. More aspects and considerations regarding the HMI are discussed in Section 4.

### 2.2. Sensor Data

The transport industry benefited from the spread of digitalization in numerous ways. Digitalization provides a way to incorporate an enormous amount of data into decisions. Applications with more data usually make better decisions compared to low-information cases, regardless of the application’s scale or scope. The availability of the sensor also drives digitalization because digitalization provides a simple, reconfigurable, and structured way to process sensor data. Due to the availability of relatively cheap sensors and processing units, sensor data usage became essential in the transport industry. Of course, V2X can also be considered a unique distributed sensor infrastructure due to the continuously disseminated large volume of information it provides.

#### 2.2.1. Vehicular Sensors

The automotive industry has sought sensor information from the beginning of the industry. Monitoring various engine parameters was always essential to safely operating the vehicles. Initially, this was solved via analog sensors. The most important sensors included the oil temperature level, the speedometer, and the odometer, among others.

In the 1970s, vehicle manufacturers started to use engine control units to manage the engines electronically. These units needed feedback from various sensors from the power train. These efforts resulted in increased fuel utilization efficiency, which could lower fuel consumption and increase power. The spread of ECUs also resulted in enhanced diagnostics which utilizes the sensor measurements to provide debug information for the mechanics. On-board diagnostics implements the vehicle’s self-diagnostic and reporting capabilities. This technique was first used in 1968 in production.

From the 1980s, various safety-related applications were also put into production. Such systems included ABS and ASR, and ESP from the next decade. These systems monitor multiple parameters of the wheels and control the acceleration or deceleration to ensure the vehicle’s safe operation. The ABS avoids the blocking of the wheel so that the vehicle remains controllable even with hard brakes on slippery roads. The ASR avoids the wheel slipping, while the ESP is responsible for the vehicle’s stability, even during dangerous maneuvers.

**Figure 4:**
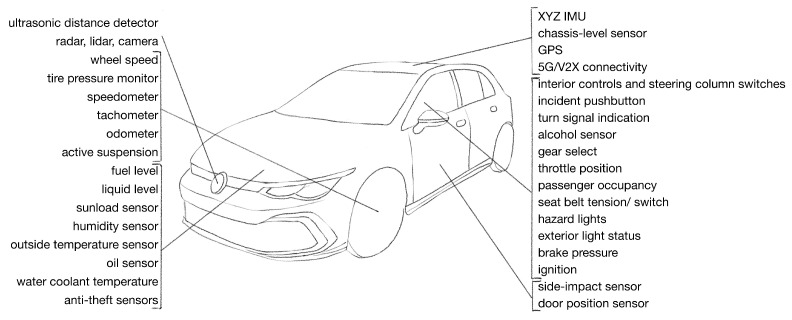
Sensors in a vehicle.

In the 2000s and 2010s, most analog sensors were replaced by digital ones. Digitalization helped to structure the data and configure the components based on various sensors. In these decades, digital sensors and processing units were cheaper. The suppliers and the OEMs started to adapt software-defined operation methodologies. This approach requires a lower number of hardware component types to be designed and tested. Still, eventually, the component’s behavior can be different in various vehicles depending on the configurations. In the 2000s and 2010s, vehicles were equipped with many sensors. These sensors may be used for efficiency, safety, or comfort features. The vehicles were also equipped with environmental sensing equipment, such as cameras detecting the lanes, radars seeing the surrounding vehicles, or parking sensors searching for possible obstacles [75]. The available sensor information also facilitates ADAS use cases. Nowadays, vehicles are also connected to rely on sensors shared by third-party road users or the infrastructure operator. This may include navigation data or V2X data. V2X can be used as a sensor to provide low-latency and high-quality data for the vehicle’s internal systems about the environment of the vehicle. Figure 4 represents a modern vehicle and some of its numerous sensors.

In the future, a higher level of automation is expected to be reached. This increases the demand for high-quality, low-latency information about the surrounding of the vehicle, which implies that more and better sensors are needed to be installed in the vehicles.

#### 2.2.2. Infrastructure Sensors

**Figure 5:**
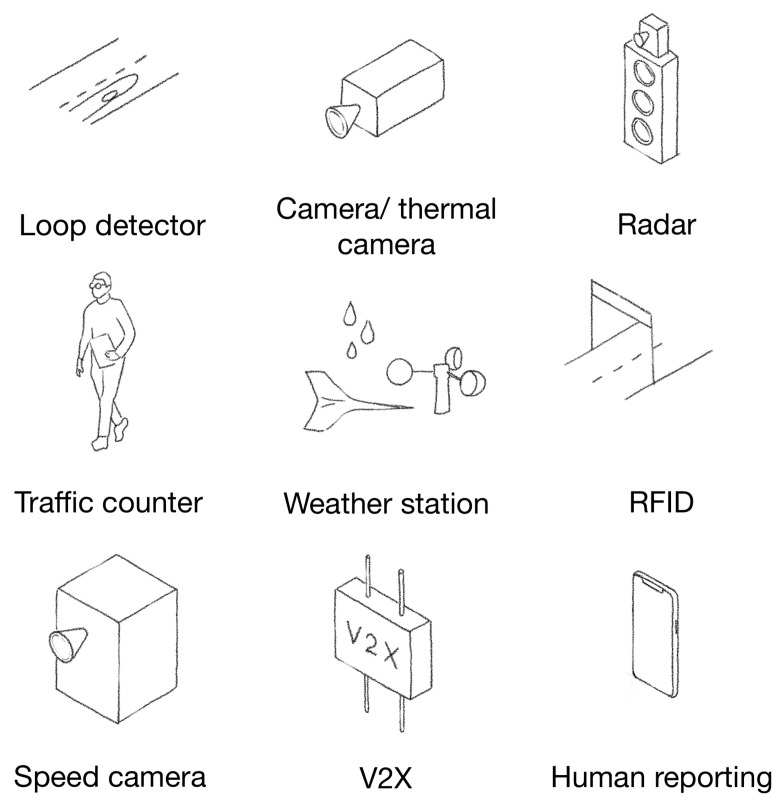
Sensors used by the infrastructure.

While vehicle usage became widespread worldwide, the road network had to be expanded to serve the increasing demand for motorization. The growing size of the road network raised various challenges. Besides legal and other obligations, a vast road network was becoming more difficult to oversee and monitor. Thus, sensors were implemented and deployed to face these challenges. Some of these sensors are depicted in Figure 5.

From a road operator’s perspective, knowing a specific road’s state is a challenge. A meteorology station can help to describe the weather situations in the surrounding stations. In the winter, this helps to organize road cleaning activities. The counting of the traffic is also a critical use case. To count the vehicles, loop detectors can be built into the road surface. Loop detectors can also be used to detect traffic jams. Cameras or thermal cameras can be used to monitor traffic in real time. The operators can detect stopped vehicles or other anomalies with these techniques. Radars can be used to detect road occupancy or upcoming vehicles, which can be used to optimize traffic light sequences. V2X can also be used as a sensor, as we already expressed.

Besides monitoring and detection, sensors can also be used for enforcement. Speed cameras can be deployed to enforce speed limits, and cameras can be used to punish red light violations or wrong-way driving. Tolling can also be automated via sensors. A camera can detect the license plate or a vignette, or an RFID reader can read the tag of a vehicle.

Intelligent transportation systems cover massive systems which aim to manage and increase the efficiency, coordination, and safety of transportation systems based on real-world data (typically from sensors [76]). These systems solve various problems, including tolling, law enforcement, and traffic management. In order to implement a more efficient transportation network, they use sensors to detect various events or take measures. Based on the sensory data, these systems can indirectly or directly influence traffic, for example, via variable road signage. C-ITS refers to cooperative ITS, which is the next step in the ITS evolution. C-ITS incorporates communication into problem resolution, opening up many new use cases. C-ITS enables the use of V2X as a sensor source and information distribution. With this next step of digitalization, many errors, such as false road sign detection, can be optimized.

### 2.3. V2X Protocols for Cooperative Perception and Information Sharing

In the ITS architecture, as shown in Figure 3, protocols of the facilities layer implement features that essentially make V2X a unique sensor for autonomous driving. Various messaging services help to transfer information from road users or infrastructure devices to enhance the perception capability based on highly cooperative behavior. The first in the list of ITS facilities (summarized in Table 1) that makes V2X an infrastructure of cooperative perception and information sharing is the cooperative awareness (CA) basic service [44]. Its message type, CAM, can be considered one of the essential V2X information carriers, as its task is to provide a relevant status description of a specific vehicle in traffic at the time the message is sent. Of course, it also provides a general description of the sender road user, which does not change during transport (attribute information, such as the type of vehicle and its physical size). As for the time-varying characterization, which can be applied to any vehicle in traffic, it includes the current position, speed, and direction, amongst others. CAM messages have many options for describing other important information. For example, if the sender is a truck, the danger of the load can be indicated, and if the vehicle is equipped with a particular distinguishing signal (e.g., an emergency vehicle), the CAM message can inform whether the sender is using its light and/or sound-based signal at the given moment. The cooperative awareness basic service periodically sends CA messages that carry containers and will be broadcasted based on specific generation rules. The mentioned containers may consist of mandatory and optional parts, depending on the vehicle type. The containers must be attached to the package at different frequencies, thus minimizing the package size. CAM messages are sent with a frequency between 1 and 10 Hz, following certain generation rules, which aim to keep information from entities fresh, avoiding channel overload.

The decentralized environmental notification (DEN) basic service and its messages are used to notify the receivers about events and changes in the traffic environment. Although the most crucial goal of C-ITS systems is to make the roads safer, traffic accidents must always be expected; the DENM message protocol [45] also characterizes such events. An environmental event can also be a stationary vehicle or a road renovation on a given section, which may mean the partial or complete closure of the road section or specific lane/lanes. An event can be characterized by its type, the time of its detection (or even its disappearance), its position, and many other event-dependent optional descriptors. For example, in the case of road renovation, the DENM message can inform about possible regional speed restrictions or restrictions on the type of vehicles passing through (such as in cases when only a certain set of target vehicles can pass through the section). It is important to add that the protocol also allows the description of events with non-static, i.e., time-varying positions (moving events). The message’s mandatory element is the management container, which defines the event’s creation time, duration, scope, and other, not the event itself, but typically its temporal, spatial, and other messaging parameters. It also contains various optional containers that describe the triggering event. When triggering DENM, the expected end of the event must be specified, as well as the sending frequency. The originating unit then sends DENMs. If the originally sending entity does not send more DENM messages, then another entity will start sending the DENM message after a certain period of time, as defined by the standard [45]. The DENM transmission can be terminated by an entity other than the original sender.

The name cooperative perception service (CPS) [48,49] aptly captures the meaning of this protocol’s existence since its task is to share the information collected by the vehicle’s sensors (machine perception) with the other C-ITS entities in its environment. In the future, this could be the basis of a service that dramatically helps the realization of self-driving vehicles. CP messages, such as CAMs, are generated at a frequency between 1 and 10 Hz. Its structure is similar to the DEN message in that there is a management container and many optional containers in addition. This protocol is a relatively recent development; currently, only a few implementations exist, but this protocol has enormous potential as it can indirectly describe important information about the road infrastructure, such as the occupancy, and it is also able to describe vehicles and objects that do not have V2X radio capabilities, thus practically extending the detection capabilities of V2X. The CP service can also provide more information than the legacy monitoring systems implemented in the infrastructure (e.g., camera systems) since CP brings all compatible vehicle sensors inside the area into the V2X sensor system as a potential perception device.

The MAPEM and SPATEM (signal phase and timing extended message) are V2X protocol messages broadcasted by the infrastructure elements (RSUs) based on the SAE J2735 standard [50], supplemented by a suitable station identifier. The MAPEM messages [47] describe the geometry of intersections. This includes the width of the traffic lane, the painting between the lanes (i.e., lane changing options), the overlapping of the lanes, the passing and turning directions, the speed limit for the given lanes, the types of vehicles allowed to use the lanes (e.g., an exclusive bus lane), the painted pedestrian crossings, and bicycle lanes. In fact, they contain almost completely static data elements, so in practice, they are helping receiver road users to discover certain details of the road infrastructure. This information typically does not change over time for a given intersection. However, any traffic changes affecting a road section or intersection will be communicated through MAPEM messages. The SPATEM protocol [47] deals with precisely sharing traffic control light status at intersections. While MAPEM messages primarily focus on traffic geometry and traffic rules, SPATEM determines which traffic lane is controlled by which traffic light. In the description of the states, in addition to normal operation, special situations are also defined, for example, the complete absence of a signal light, in which case the general crossing rules apply. These messages are sent at a configurable sending frequency.

The infrastructure to vehicle information message (IVIM) messages, as the name suggests, provide informative information published by the transport infrastructure to vehicles on the roads. In each case, this information is assigned to specific traffic regions. For example, in the case of a speed limit information message, it must always be indicated which road section it applies to. In the case of multi-lane traffic, the direction can also be specified separately. The IVIM protocol [47] typically provides restriction information, such as messages about speed, weight, size, and emission limits, but of course, many other various road signs can be transmitted via V2X communication based on this standard. Although it is possible to interpret the restrictions for all passing vehicles by default, the optional fields of the message allow for a detailed definition of the affected vehicles, especially concerning the cargo of trucks. This message type is based on the corresponding ISO specifications [46], extending it with a station identifier. The IVIM protocol defines zones for the validity of signals. For example, it separates the relevance zone (i.e., the zone where the given signal is valid) from the detection zone, where vehicles only have to notify their drivers. Of course, there can be more than one relevance zone. In addition to the broad signal definition, IVIM is also suitable for sending a signal via text. Road operators can also use it to dynamically introduce speed limits, emissions, and other restrictions and important information.

The signal request extended message (SREM) and signal request status extended message (SSEM) messages [47] are based on the J2735 standard [50] used for traffic-dependent control of traffic lights as a part of the traffic light control (TLC) service [47]. SREM is a request from the vehicle to the infrastructure, and the infrastructure informs about its assessment in an SSEM message. It is understandable that, in this context, the request from the vehicle is to turn the traffic lights of the affected route green, thus ensuring uninterrupted passage or green wave. Since many vehicles may have such requests at the same time interval, it is up to the infrastructure to fulfill them according to the exact prioritization. With the help of its effective use, major traffic jams can be avoided (as long as it is optimized to try to reduce the longest congestion in the region in terms of the number of vehicles), unnecessary waiting times can be reduced (e.g., green can be ensured continuously at an intersection, if there is no cross-crossing traffic at all), traffic in larger regions can be optimized (so it does not necessarily take a single intersection or road section into account for decision-making) and the passage time of high-priority vehicles can be minimized (e.g., in the case of priority convoys or emergency vehicles).

The vulnerable road user awareness message (VAM) protocol [77] describes those participants in traffic that are not classified as vehicles. They are, therefore, classified as vulnerable road users (VRUs), such as cyclists and pedestrians crossing the road, who are exceptionally easily injured in a collision with a motor vehicle. This message type is expected to be implemented based on the SAE J2735 personal safety message standard [50]. The message can also describe other entities that are present, such as road construction workers, human-drawn or physically powered or animal-drawn, slow-moving vehicles (such as rickshaws, pedicabs, or horse-drawn carts) in traffic, police officers manually directing traffic, and even stray wild animals. The protocol determines the position of the VRUs, the accuracy of their current position, speed, acceleration, the direction of movement, the trajectory they have traveled so far, and their expected future route. There are several options for defining entities. It is possible to specify whether someone is crossing the designated pedestrian crossing sitting in a wheelchair or riding a skateboard, what task the person on the road is performing (e.g., preparing to tow a vehicle, fighting a fire, or catching a wild animal), and even whether the person crossing the road is currently on the phone or listens to music (so the attention span is limited). The VAM message can be extremely beneficial in accident avoidance because it provides an opportunity to receive the exact position and other information about vulnerable road users, which we could not describe only with CAM-type messaging.

The maneuver coordination message (MCM) [78] and platooning control message (PCM) [79] messages were designed to control vehicle convoys, but they were not yet standardized at the time of this study. In their function, they enable several vehicles to change lanes and overtake in a coordinated manner and reunite broken convoys. Moreover, the application of these protocols is primarily considered for connected and autonomous vehicles (CAVs): visualization of the information they carry or operate with is not relevant for human driver use cases.

### 2.4. Relevant V2X Use Cases

This section presents the introduction of the main V2X use case groups of early, first-phase (Day 1) C-ITS deployments to highlight relations of data collected/exchanged with the above-depicted protocols in the extensively cooperative C-ITS ecosystem and their possible visualization considerations in implemented applications, divided into five main categories [51,80,81]. Next-generation (Day 2, Day 3, and beyond) use cases will not only differ from Day 1 in the applied types of Facilities protocols (a good number of those V2X messaging and communication schemes are already available in Day 1 use cases), but significant differences will be found in the requirements and reliability conditions mostly [82]. That is why we focus on Day 1 in this article and mention only a few use cases of the later phase of the V2X evolution. Table 2 summarizes the identified ITS service groups and examples for relevant use cases discussed in the following paragraphs.

#### 2.4.1. In-Vehicle Signage (IVS)

Mandatory road traffic regulations for a given road section or region are typically communicated to the driver by using static/variable traffic signs, highway information boards, and road surface markings. Legacy signboards and paintings are more static (so they cannot be changed arbitrarily in real time). In contrast, the content displayed by information boards and variable signs can be changed at any time. Still, their use is limited to a few typical areas (e.g., warning about congestion). The characteristic feature of all three is that the information they carry reaches the vehicle’s driver at the earliest within the limit of the human line of sight, and the installation location with weather conditions limits the transmission of information. However, with the help of V2X, helpful information can be transmitted to the vehicle in IVS use cases [81,82] and displayed via the appropriate HMI regardless of the installation or visibility limitations and even significantly earlier than the arrival at the place of use. Besides not being affected by possible bad visibility conditions, an additional advantage of this type of display is that there is not a highly limited, speed-dependent time window in which the user can extract the information. In practice, this last statement means that the faster a vehicle moves, the less time the driver has to process the information carried by the signs visually. This can cause problems, especially on motorways, where even longer text can be displayed in addition to a speed limit value or a traffic pictogram. The information provided in the vehicle makes it possible for the vehicle’s driver not to miss any information essential for complying with the traffic rules, thanks to the more permanent displaying method. Since the traffic paintings on the surface of the roads provides more immediate data, and in the case of regional information, it is redundant and repetitive (e.g., a discontinuing lane or bus lane), IVS solutions can play an essential role in the IVIM-based transmission of information originally carried by legacy static and dynamic signs.

#### 2.4.2. Hazardous Locations Notification (HLN)

One of the unfortunate consequences of driving a car is that dangerous situations can arise on the roads at any time, which can affect the safety and efficiency of traffic. The primary purpose of C-ITS systems is to prevent possible collateral accidents that arise from risks. Although an emerging emergency can mean personal injury or even the loss of human life (e.g., a major traffic accident), accidents based on these can often have more severe consequences. However, it should be noted that many dangerous situations are problems only at the level of indirect complications and, thus, sufficient information about the original event can even drastically reduce the chance of accidents occurring. Another vital task of C-ITS solutions is that the information provided by the infrastructure can safely increase the efficiency of traffic passage in the emergency region. Timely delivery of relevant information to motor vehicle drivers can enable them to search for alternative routes, thereby avoiding the dangerous area and the possible traffic congestion that may arise around it. If this is not possible (that is, if the traffic topology does not allow avoiding the given area), the vehicle’s driver can prepare in time for the risks of an emergency. Use cases in the Hazardous Locations Notification major group [81,82] use DENM (sometimes CAM) protocols for the information dissemination mechanisms of the above functionality.

#### 2.4.3. Road Works Warning (RWW)

There are many reasons for maintenance work on roads and other types of traffic interventions. This includes repairing damage to the road surface, transforming traffic junctions and road sections (e.g., creating a roundabout at an intersection), and removing snow and ice. Their common feature is that the schedule of the works can be planned in advance, and their completion is usually subject to a limited time limit, but they have not considered an emergency intervention. Sometimes the duration of the restrictions they implement (e.g., lane closures) can be one or more orders of magnitude longer than in the case of an emergency activity (e.g., works lasting weeks or months). Temporary traffic redirection will be implemented for larger-scale activities, and the long-term traffic topology may change when completed. Road maintenance works typically impact both the safety and efficiency of traffic. In terms of closures, they are similar to emergencies. However, more prolonged closures allow for more straightforward adaptation due to the less unexpected nature of traffic restrictions and through the strategic planning of comprehensive traffic diversions, thus representing a lower risk of accidents and deterioration of transit efficiency compared to sudden events. Nevertheless, adequate information can significantly improve both aspects, where visualization of received road works warning reports is crucial to enhance drivers’ awareness optimally. RWW use cases [81,82] solve this by relying on DENM protocol messaging services to share relevant information between road users and/or the infrastructure (timely and precisely).

#### 2.4.4. Signalized Intersections (SI)

Traffic signals operating at intersections are considered one of the most important means of regulating the movement of vehicle traffic in cities. The traffic lights are coordinated with each other in terms of safety and efficiency. It is a severe safety consideration: for example, in the case of intersecting traffic lanes, the traffic system only starts the other after stopping one, and sometimes there are even a few seconds between such switches. At the same time, traffic lights must be synchronized efficiently to minimize the periods when no passing traffic is allowed. Moreover, the traffic load on different road sections must be equalized by design. The efficiency aspect of the use cases included here is also cost-effective since vehicles waiting for the green sign consume resources, which in the case of fossil fuels also entails severe local environmental considerations. The purpose of the signalized intersections use cases [81,82] is for C-ITS solutions to provide safer and more efficient crossings at intersections compared to current traffic systems, which globally has an impact on other sections of the traffic as well. Another advantage of the application of MAPEM, SPATEM, SREM, and SSEM protocols in V2X-aware intersections is that the schedule of traffic lights can be dynamically adjusted in special cases (e.g., in the presence of an emergency vehicle), and in addition, C-ITS systems can actively take into account the traffic currently affecting the given intersection, such achieving dynamic and adaptive behavior. This requires clear and timely visualization of the received information (warnings, speed advisory, intersection geometry, signal phase, timing data, etc.), which is essential for human drivers to follow suggestions and avoid dangerous situations.

#### 2.4.5. V2V-Specific Use Cases

We have already touched on some of the V2V use cases in the previous groups despite the fact that those presented infrastructure-focused service types mainly rely on V2I/I2V communication patterns. However, V2V applications in the groups mentioned above are still usually tied to the infrastructure in some way, or at least to the duties and functions of the road operator (e.g., in the case of an HLN Emergency vehicle approaching, typically it is the road manager’s vehicle that starts warning message dissemination to avoid accidents). Here, we briefly present some of the most important representatives of Day 1 use cases, which do not belong to the groups discussed before, operate exclusively in a V2V context, and can be considered independent of the infrastructure and the road manager in this respect [82].

In the Dangerous Situation Warning for Electronic Emergency Break Light use case [52], vehicles rely on DENM to communicate with their immediate surroundings in a V2V context if the driver has performed an emergency braking maneuver. The emergency braking is either detected based on the threshold value for braking deceleration or based on the internal signaling of the vehicle. Based on the detection, the triggering vehicle notifies its V2X subsystem. The V2X subsystem populates the DENM using the available data. The trace is filled with the path history of the triggering vehicle. The triggering vehicle schedules the DENMs, which are repeated over the air accordingly. The receiver vehicle interprets the message, and uses the HMI to inform drivers about this potentially dangerous situation.

The exchange of impact reduction containers [53] is triggered if an immediate and likely unavoidable collision scenario occurs. The detection of imminent and inevitable accidents may be performed based on a CAM-based message exchange. In case a vehicle detects such a scenario, it immediately sends out DENMs populated with Impact Reduction Container requests. Vehicles in close proximity respond to this request. Both the request and the response contain accurate information about the current status of the sending vehicle (including the position of the occupants, the mass of the vehicle, the turning radius, the position of the pillars, etc.). Using this additional data, the vehicles can have a better overview of the situation, including the occupants to be protected and the dynamics of the colliding vehicles; thus, the safety systems of the vehicle can reduce the consequences of the collision.

In the case of Intersection Collision Warning [83], the V2V CAM message exchange (extended with IVIM, MAPEM/SPATEM, and DENM information) can be used to achieve safer crossings at intersections, prevent collisions or reduce consequences by detecting the risk of a dangerous situation and warning the driver accordingly. In this scenario, two (or more) vehicles are approaching an intersection. The vehicles are sending CAMs that represent their current kinematic status. The vehicles run safety applications that try to detect potentially dangerous remote vehicles. Based on the CAMs of the remote vehicles, the ego vehicle is able to predict potentially dangerous situations. On such occasions, the vehicle notifies the driver, who has sufficient time to perform an evasive maneuver.

The motorcycle warning [83] use case can warn the driver about an approaching motorcycle in selected traffic situations. This is especially useful in circumstances with reduced visibility. Using CAM exchange, the vehicle and the motorcycle both monitor the distance between them, and if it falls below a critical value, the drivers will be warned through their respective HMI subsystems, so a potential accident can be avoided by braking or slowing down.

## 3. Projection-Based Light Field Visualization Technology

### 3.1. Concepts and Principles

The core idea of light field visualization dates back to 1908, to the work of French physicist Jonas Ferdinand Gabriel Lippmann [84], in which he defined integral photography—a 3D imaging technique that captures the field of light via a microlens array, where each microlens perceives the world from a slightly different perspective. The technical term “light field” was coined later by Andrey Aleksandrovich Gershun in the 1930s [85]. Essentially, as described by integral imaging, the faithful capture of our 3D world requires many-many different viewpoints and perspectives. The simultaneous and continuous recreation of these perspectives results in the parallax effect. If only the horizontal or the vertical perspective changes, then we call it either horizontal-only parallax (HOP) or vertical-only parallax (VOP) light field, respectively. The combination of the two is known as a full-parallax (FP) light field. These terminologies may be applied to both the display system and 3D content.

In contrast to stereoscopic 3D (S3D) solutions, perspectives are not assigned to the eyes of the viewer. In fact, unlike the vast majority of 3D technologies, visualization is not centered around a single viewer. Projection-based light field displays provide the content for a given area, and any number of individuals that, of course, can fit into that area, may simultaneously view the same content from the respective orientations. Again, it needs to be emphasized that the viewers do not need any viewing device (i.e., headgear or special glasses) to perceive the full extent of light field visualization.

### 3.2. General Overview of Operation

The first fundamental property of projection-based light field displays that must be highlighted is that the screen, regardless of size and display type, is a completely passive component of the system. This means that it has no internal mechanisms or any active part. It basically works as an optical transformer, built of micro-holographic patterns. The optical transformation applies to the light rays that originate from the optical engine array (i.e., projectors). If the projectors are on the same side of the screen as the viewer(s), then we call the system a front-projection light field display. Otherwise, if the projectors and the viewer(s) are on opposite sides of the screen, then it is a back-projection light field display. It is important to distinguish light field displays from multi-view displays, as the latter relies on “sweet spots” (i.e., spatially-distributed positions from which the same autostereoscopic view can be perceived), and light field visualization utilizes the entire viewing domain to provide continuous parallax.

The content visualized by a light field display is either real (i.e., camera-captured) or synthetic (i.e., computer-generated). The latter is easier to produce due to the simplicity of virtual cameras. Computer-generated contents may be a rasterized sequence of 2D images or can be rendered via ray tracing. Using 2D images corresponds to a 1D array for HOP and VOP solutions (i.e., the perspectives differ along a given direction) and a 2D array for FP. The perspectives are captured by virtual cameras, and the resulting rasterized sequence is converted by the display system. In the case of ray tracing, we can directly generate content for the system, which is generally more convenient for automotive displays. However, pre-converted image sequences may also be adequate for the investigated use cases. If we wish to capture the real world with the help of pinhole cameras, then we either need an array of cameras, e.g., the capture system of the first-ever light field telepresence system [86], or we use a single camera, but constrain the content to be static. In the latter case, either the camera moves around the scene or the scene itself rotates. In any case, regardless of capture and generation, the data are fed to the converter of the display system in order to match it to its own parameters, known as key performance indicators (KPIs).

### 3.3. Key Performance Indicators

Light field visualization has numerous KPIs [87], many of which apply to both the display and the content. Let us first review these common properties. The resolutions of light field display and content are characterized by spatial and angular resolutions. If we imagine the visualized 3D source content as an array of 2D images, again, the 1D array in the case of HOP/VOP and 2D for FP, then the image resolution corresponds to spatial resolution. If it is insufficient, then the visualization becomes blurry [88]. Note that this is not severely penalized on a perceptual level for synthetic contents [89]. On the other hand, angular resolution is critical for any type of content [90]. It describes the density of light rays. More specifically, for displays, it is the smallest angle of change that the display can achieve [91], the smallest angle between two adjacent distinct light rays, with respect to a single point on the screen [92]. For content, if we follow the approach of using an array of 2D images as input, then it is the ratio of the number of these images and the field of view (FOV), technically, the density of source views. It is indeed critical to avoid an insufficient angular resolution, as it may compromise the smoothness of the parallax effect and may also result in the crosstalk effect, which is the interference of adjacent source perspectives. The FOV is the angle, measured from the screen, in which the visualization is valid (i.e., can be properly perceived without missing light rays). Greater FOVs dictate greater angular inputs in order to maintain the angular resolution. As the perceived angular density dissipates over distance, due to simple trigonometry, these two KPIs determine the valid viewing area (VVA). In essence, greater viewing distances require greater angular resolution values [93]. Additionally, the depth of the content may also necessitate higher angular resolution [94]. For displays, we refer to the ability to visualize depth as depth budget, which extends to both directions. It is a budget, as it does not need to be fully utilized all the time.

Among KPIs that apply only to the display system, screen dimensions are highly relevant, as they scale with the projection subsystem, and the curvature of the screen affects depth and FOV. Display brightness is measured on the screen, and for such measurement, fully white content is visualized. Both brightness and contrast are crucial to the performance of the system, particularly if the environment’s illumination is not favorable (e.g., if there are strong external light sources). The refresh rate can also have an impact, yet note that it is not relevant to static visualization.

### 3.4. Limitations and Challenges

Unfortunately, with great performance comes great burden. At the time of writing this paper, projection-based light field visualization is still emerging; it has appeared in some segments of the industry, but it is yet to appear in the consumer market. This is partially due to the many challenges and certain limitations of the technology.

A quite apparent barrier is the cost of light field visualization systems. While such technology is already being considered for industrial use cases, and defense applications may easily have a sufficient budget [95], end-user devices are currently far from affordable price ranges; this also limits the access of research institutions to light field displays.

Beyond power requirements and other implementation-related challenges, light field visualization comes with immense data that is to be stored, processed, and depending on the use case, transmitted. To combat this, there are significant research efforts related to compression [96], alternate formats [97], and adaptive approaches [98].

One major constraint of light field visualization is that the 3D content needs to be finite. This means that the visualized scene cannot have elements outside a bounding space. For instance, in the case of 2D imaging, it is possible to display objects on a far-far-away horizon with appropriate fidelity. If this is attempted for light field displays (e.g., with a flat background image), then its quality will ultimately be degraded.

According to the currently available state-of-the-art research [99], interaction using light field human–computer interfaces may be outperformed by conventional ones. Of course, this does not limit the visualization itself, only a single segment of its application.

The two major challenges for future light field displays are FP visualization and super-resolution. FP imaging can be taxing, particularly due to the aforementioned data requirements. Although HOP displays are adequate for the vast majority of current use cases, FP is needed to fully encapsulate the 3D world. Super-resolution refers to an angular density at which a single point on the screen may address one pupil with at least two distinct light rays. Achieving this goal may enable the observer to focus on the visualized content (just as in real life). Otherwise, the focus of the eyes is limited to the plane of the screen.

### 3.5. Recent Research Efforts

Recent works on light field technology cover the topics of angular enhancement [100,101,102] (which aims to achieve super-resolution), image enhancement [103,104,105] (which is also often named super-resolution, and thus, the terminology of the other one is becoming “angular super-resolution”), saliency detection [106,107,108], light field rendering [109,110,111] and reconstruction [112,113,114], microscopy [115,116,117], camera animation [118], video streaming [119], objective quality assessment [120,121], perceived quality [94,122], and many more. Every single experiment that studies the projection-based light field visualization with the involvement of human individuals is summarized in a recent work [123], along with a thorough analysis of future research efforts, and an up-to-date survey [124] investigates the associated methods. Super-resolution as a form of enhancement does not benefit automotive systems in the context of V2X, as the content is expected to be synthetic. However, the angular super-resolution of the display system holds immense value, as it enables the human eyes to change focus between portions of the content with different depth levels. Regarding subjective and objective quality assessment, while the first one helps to bridge the gap between system performance and user needs, the latter compensates for the general shortcomings (e.g., the process lengths) of user tests in the system evaluation. Additionally, objective metrics provide protection from the different forms of cognitive bias that may influence subjective studies (e.g., the labeling effect [125,126,127]).

### 3.6. Use Cases

There is a great number of potential use cases for projection-based light field visualization. This includes—but is not limited to—industrial use cases such as prototype review and resource exploration, medical imaging, telepresence, training, education, gaming, home entertainment, cinematography, digital signage, air traffic control, defense applications, exhibition of cultural heritage, and—last but not least—automotive use cases. Additionally, recent technological advancements, such as the modular, any-size, any-aspect, any-shape, and any-surface 3D light field LED wall [128], shall provide immense assistance in the re-imagining of current approaches. Effective, efficient, and user-centric deployment of the technology fundamentally depends on considerations related to potential degradation archetypes [25]. In the remainder of this section, automotive use cases are discussed in detail.

#### Automotive Use Cases

**Figure 6:**
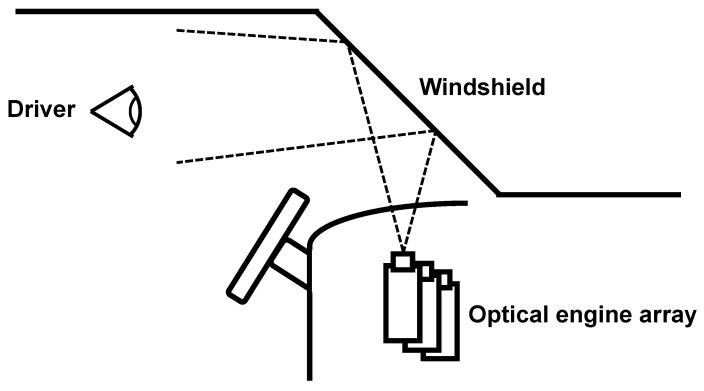
Concept of an onboard light field visualization system [129].

In the general case of automotive utilization of projection-based light field visualization, the windshield of the vehicle is used as the passive screen, which transforms the light rays that originate from the optical engine array. The concept of such an onboard light field visualization system is shown in Figure 6, based on patent WO 2005/117458 A2 [129]. It needs to be highlighted that the windshield in the figure has transparent holographic screens attached to it.

First of all, note that such visualization is primarily focused on the driver. It is technologically feasible to consider the front-seat passenger as well, but for the scope of this paper, which is built around V2X communication, the driver is the sole observer of the use cases.

In the scientific literature, the publication by Gabbard et al. [130] provides a review of the challenges and potentials of such automotive applications. The case study of Charissis et al. [131] targets hazards associated with adverse weather conditions (i.e., risks due to low visibility). The simulation trials with 40 test participants evaluated the visualization of sharp turn notifications. Bark et al. [132] particularly focused on navigation applications. The paper of Rao et al. [133] analyzes the influence of such HMIs on the design of the in-vehicle electric and electronic architecture, which is a rather essential implementation-related consideration. The work of Yöntem et al. [28] is not only notable for its comprehensive overview of state-of-the-art solutions but also for proposing and implementing a distributed display system (DDS) that utilizes the entire windshield. The prototype exhibits various use case contents simultaneously (e.g., car approaching, speed limit, turn direction). Moreover, the authors emphasize that any window in a vehicle may be used as a display, not just the windshield. A summary and comparison of the scientific literature on immersive windshield interface technologies are provided in Table 3.

**Figure 7:**
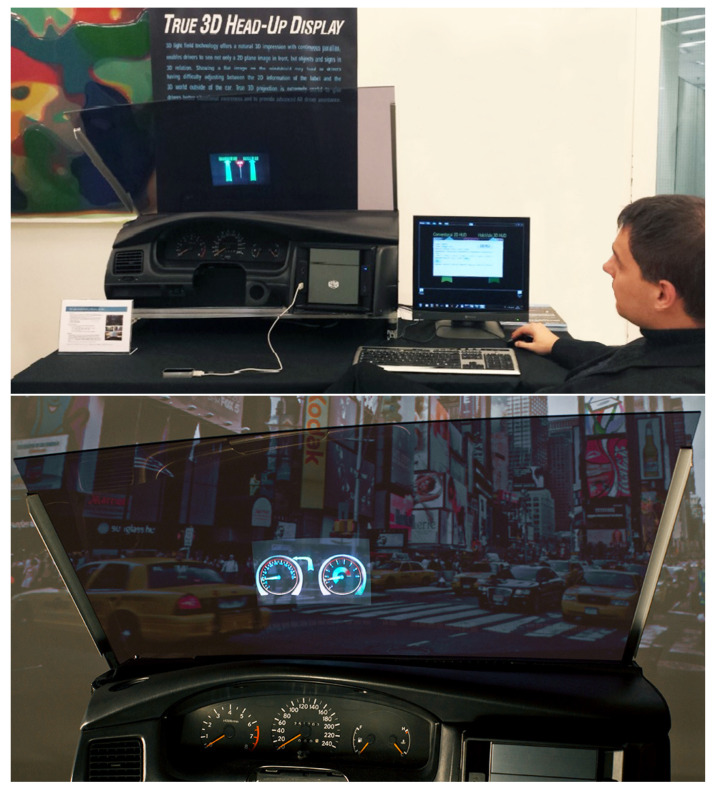
Exhibition of the first automotive light field prototype; © Holografika.

The automotive prototype of Holografika was created in an industrial context, and it was never published in the scientific literature. It is important to distinguish that while the concept presented in Figure 6 relies on a transparent holographic layer added to the windshield, the prototype at hand, which is the world’s first automotive light field system, does not require such, and handles the technological challenges via calibration. A demonstration of the prototype is shown in Figure 7.

Additionally, it is important to highlight that the solutions addressed by this paper do not rely on eye-tracking. For example, the work of Lee et al. [19] proposes a light-field-based 3D HUD that uses eye-tracking. Furthermore, there are immersive HMIs in the scientific literature that show promise for the automotive context, such as the light field prototype of Duarte et al. [22], yet are not designed particularly for the investigated utilization.

Let us now review the technical and KPI-related requirements and considerations of such a system. One may first think of power consumption. Indeed, large-scale light field systems have massive power requirements, as not only a great number of high-performance optical engines must be powered, but also the associated computer clusters as well. In automotive use cases, power is less of an issue, as the optical engines only need to address a relatively small surface, in comparison to high-end light field displays, which may have screen width in the order of meters. Additionally, no conversion is required if the content is fully synthetic and is generated by a small onboard unit, the input to which is provided by V2X protocols. Such an onboard system is also exhibited by the prototype of Holografika, as shown in Figure 7.

Again, neither power nor data pose significant challenges. Yet the KPIs of visualization are still to be considered. As the visualized content is synthetic, spatial resolution can be rather low. The same cannot be said for angular resolution, as parallax-related issues may visually irritate the driver and, thus, may endanger all the passengers and other participants in the traffic. Therefore, the crosstalk effect must be avoided; hence the angular resolution should normally be 1 degree or better. Note that “better” in this scientific context refers to smaller degrees (e.g., 0.5 degrees is better than 1 degree). However, according to the calculations of one of our recent works [143], if the appropriate angular resolution is defined as

(1) $$Angular\;\;resolution=arctan(\frac{Average\;\;interpupillary\;\;distance\times p}{Maximum\;\;viewing\;\;distance}),$$

where *p* is a use-case-specific variable set to 0.7, then even a one-meter viewing distance can be satisfied by an angular resolution of roughly 1.5 degrees. Fine-tuning of the *p* value is recommended for special-purpose vehicles, where the distance between the windshield and the driver’s pupils may deviate from the commercial averages.

The horizontal FOV does not pose a challenge (as the potential viewing angle interval does); technically, the entire VVA can be easily narrowed down. A small, well-defined FOV is beneficial in multiple terms of projection performance.

On the other hand, the depth budget is a more divisive matter. Basically, for visualization applications that aim to perceptually “draw on the road” (e.g., directions), more depth is required. Evidently, more depth necessitates higher angular resolution. Luckily, as written before, the need for angular density is slightly mitigated via the constraints of the viewing conditions.

Brightness and contrast are absolutely essential to the successful implementation of such a system. Automotive use cases are even more difficult to satisfy than digital signage. Similarly, in the case of digital signage, the autostereoscopic 3D billboard needs to be properly visible during the sunniest of days. However, while the potential issues of digital signage result mainly in monetary problems (i.e., the commercial content does not reach the consumers), any such issue in the case of automotive use cases may risk the safety of the participants of the traffic. Additionally, during practical utilization, the performance of such a light field interface should be adequate regardless of the cleanliness of the windshield. In essence, while laboratory-based prototypes enjoy the eternal sunshine of the spotless windshield, brightness and contrast must endure realistic conditions for the sake of usability and, thus, safety.

Finally, refresh rate can be considered a rather irrelevant KPI in this context. Basically, V2X information can be conveyed without the need for content animation. Even if there is some animation, its frame rate is not even remotely critical to the effectiveness and efficiency of the use case.

All KPIs considered, such projection-based solutions may vary a lot when it comes to objective capabilities. Due to the aforementioned properties of automotive use cases, adequate, function-focused implementations are feasible and are significantly less expensive than general-purpose light field displays; while at the same time, it is, in fact, possible to create resource-demanding, high-end systems. The approach of the well-known CEO of General Motors, Alfred P. Sloan, was that there is “a car for every purse and purpose”. In terms of projection system design, the 1925 slogan of Sloan may be applicable in this context as well.

## 4. Recommendations for V2X Information Visualization

The first major consideration for V2X information visualization via light field technology is that the driver must not be over-encumbered with information. This means that the available visualization space must be limited both in terms of spatial fill and the number of different information pieces. Based on this consideration, only the most vital, essential V2X information should be visualized.

One of the greatest advantages of such visualization is that the driver does not need to divert attention away from the traffic. This is particularly beneficial for safety-related, time-critical messages. For instance, in case of an imminent collision warning, without such windshield-based information relaying, the driver would need to look at other sources of V2X warnings. Since such a scenario is particularly a time-sensitive one, losing even the shortest amount of time to switch between the observation of the traffic and the other information interface could be extremely counterproductive, as it would carry the potential to constrain the driver’s ability to mitigate the situation. Therefore, one of the greatest priorities in such utilization of light field technology is to visualize safety-related warnings. Technically speaking, numerous types of data can be adequately displayed in other equipment, such as an onboard unit or a smart device.

The visualization of information can be based on the location of the source; on the distance of the relevant traffic area. For example, let us say that there is a segment of the road infrastructure that is affected by roadworks. On the one hand, the notification about the roadworks is not an urgent message if it is situated kilometers from the vehicle’s current position. On the other hand, there may be a case where the later portions of the road infrastructure limit the possibilities for detours. In any case, such a warning has lower priority than many other safety-related messages. Basically, we can recommend that such information is only visualized on the light field screen if the vehicle approaches the last convenient portion of the road infrastructure where a detour is possible. In any other case, this information can be accommodated by other devices.

If there are no warnings to display on the screen, there are many protocols that could be excessively useful for the driver. For instance, green light optimum speed advisory (GLOSA), a specialized use case of the signalized intersection group, provides a recommended speed value to assist the driver in achieving a series of green lights in the traffic. Advising a specific speed interval on the light field screen can improve the efficiency of the driver’s trip. Additionally, this has environment-related and financial benefits since the lack of stops and slowdowns reduces the consumption of fuel (regardless of the fuel type).

In the case of IVS messaging, visualizing specific traffic-related messages on the light field screen is not a priority. However, we do recommend that if no warnings are present, then a notification is displayed about the presence of a new IVS message that can be read elsewhere (e.g., on the onboard unit). Such messages can be longer, and many of them cannot be properly represented by pictograms and icons. For example, there can be an IVI message that notifies drivers that the road to the airport will be closed during the weekend. As this message is far too complicated to be displayed on the light field screen (i.e., such a message can be particularly long and complex, diverting the attention of the driver), only the presence of such an incoming message should serve as a basis of notification.

Graphic indicators should have a priority in visualization. In essence, texts longer than one or two words should not be displayed on the light field screen. Moreover, the visualization of text should be limited in depth to provide the best possible perceived quality.

For special-purpose vehicles, such as vehicles of law enforcement, it is possible to request passages in intersections in which the traffic control lights would not permit it. The SREM message requests a passage, and the SSEM message provides a notification regarding the passage. In this usage context, we recommend that the brief content of SSEM messages (i.e., a “request accepted” or a “request denied” message in a pictographic format) are displayed on the light field screen, as they directly affect the passage of the vehicle.

A specific application of V2V-specific use cases is lane-changing assistance. The generated messages of such communication can be warnings about overtaking. For instance, if a driver uses the turn signal to initiate an overtaking, but this is in contradiction with the incoming traffic information (e.g., there is a vehicle out of the line-of-sight of the driver in the other lane), then we recommend that a warning message is relayed to the driver on the light field screen, in order to avoid such traffic hazard. This generally applies to VRU-related warnings as well.

The V2X applications considered by this section primarily belong to Day 1 services. Day 2 and beyond are out of the scope of this current work; however, the generalization of these guidelines applies to those as well.

## 5. Discussion and Implications

Light field visualization of V2X information can help human drivers to better understand their environment by providing a more comprehensive view of the current traffic situation, presenting a broader picture of the road infrastructure’s actual status, for example, in an intersection or a parking lot, including the position of other vehicles, pedestrians, traffic signals, and other objects. This can help drivers make better decisions and reduce the risk of collisions, allowing them to detect and respond to potential hazards or traffic situations more quickly and accurately.

V2X applications can benefit from light field visualization because it allows for realistic 3D views of objects on the HMI side, providing a more immersive experience and a more realistic view of the environment. This can be particularly useful for IVI applications when the HMI must efficiently represent the received information. For example, light field visualization can show the applicable traffic rules accurately on the road, knowing the exact spatial scope of the restrictions. It can also show textual adversary information shared by the road operator so that the driver can read the critical information even if the roadside displays were missed. In HLN use cases, the driver can more accurately and quickly identify a scene’s actors, objects, and features. This can help improve the accuracy and speed of situation recognition and object detection, tracking maneuvers and tasks on the road, and identifying and filtering false positive warnings. V2X messages are able to communicate the exact location and type of the event cause. The path towards the hazardous location is also shared along, so the driver can be informed well ahead, not just about the upcoming danger but also about the path towards the affected area (e.g., via highlighting the path to the dangerous area, which should be, naturally, avoided). In the case of RWW, light field visualization techniques, for example, can enable presenting complex traffic scenarios in 3D, allowing for a better understanding of the situation and how different vehicles interact with each other. Additionally, it can help identify potential safety issues and provide a more detailed overview of traffic conditions. V2X messages contain relevant metadata about the road event, such as road closures, speed limitations, etc. The proper representation and communication of these data to the driver can greatly increase situational awareness, especially in scenarios where the visibility of the scene is hindered by the weather, large vehicles, or other factors. Light field visualization can also be used to provide more accurate and timely warnings to drivers, allowing them to make better decisions in real time. Unlike most current solutions, which only warn the driver about the fact of the danger, light field visualization may also show the spatial properties of the upcoming risk to the driver, so the driver can better understand the actual traffic situation. Moreover, it can help to improve the accuracy of representation and reliability of the usage of data collected from V2X/C-ITS systems, allowing for more effective and precise responses during any type of traffic situation.

There is indeed a myriad of information that could be visualized on the light field screen of a vehicle. However, it needs to be highlighted that this article particularly focuses on V2X communication.

Regarding the visual utilization of the screen, it is a discussion among experts that only the lower half of the screen area should be used for such content. By the lower half, we refer to the portion below the horizon. The advantage of this approach is that information has a lower perceptual impact on the traffic in this case. At the same time, this could be a disadvantage in a traffic context with lower-quality road infrastructure (e.g., it would be harder to avoid small potholes).

As for the actual contents, graphic visualization is most definitely recommended. For this, the ISO/TS 14823 standard already defines a graphic data dictionary. In future work, it is worth addressing the adaptation of the standardized graphic media in an autostereoscopic 3D context.

V2X protocols may also provide precise information regarding the aforementioned issues of the road infrastructure and hazardous objects (e.g., a tree branch that landed on the road after a storm). Based on accurate location data, it is possible to visually indicate such issues and objects on the road, increasing traffic safety. V2X messaging enables information sharing about other road users and perceived objects as well (e.g., via CAMs and CPMs, respectively). With the help of light field visualization, potentially dangerous objects can be communicated to the drivers, even if the visibility of the object is blocked by another vehicle or a building. In the future, V2X will also support sharing the intention of the road users so that the future path of the surrounding vehicles can also be represented to the driver.

In the context of this paper, individual vehicles are discussed. However, there are already V2X use cases where vehicles travel in formation. This is called platooning, during which it is now possible that a human driver operates only the platoon leader (i.e., the first vehicle), and the other vehicles follow. Since platoon vehicles are often larger trucks, their windshield sizes enable larger light field screens. This is quite relevant, as it would be most beneficial for a platoon leader to efficiently receive real-time information regarding the vehicles of the platoon. Follower vehicles in a platoon are usually seated with a driver as well. In these cases, the workload of those drivers is reduced, but in certain situations, they have to take over the control of their vehicles. During the takeover, the drivers have very limited awareness of the traffic situation because the preceding truck blocks a huge part of the view. Light field visualization can help build up awareness through real-time visualization of the other trucks, their speeds, tracks, surrounding objects, or possible upcoming dangerous locations.

Additionally, it should be highlighted that the angularly selective nature of projection-based light field displays enables completely different content for users who observe the screens from different viewing angles. Through such, it is possible to visualize V2X-related information to the driver, even on the entirety of the windshield, while the person in the passenger seat engages with unrelated contents, such as multimedia or interactive applications. Again, it is possible for both the driver and the passenger to utilize the entire screen simultaneously without interfering with each other.

Finally, let us have an outlook regarding autonomous vehicles. Scottish-American financial journalist B. C. Forbes—the founder of Forbes magazine—once said that “a business, like an automobile, has to be driven, in order to get results”. While we are rather far away from reaching the point of self-driving businesses, the long-term evolution of personal transportation via automobiles gravitates toward autonomous solutions. In such cases, the windshield could serve multiple purposes. When the vehicle is in a fully autonomous mode, the light field screen could function, for instance, as an onboard entertainment system. There have already been discussions among experts to facilitate video calls on such automotive displays. With the future emergence of light field telepresence, for which the first operational dedicated prototype was introduced in 2018 [86], as mentioned earlier in the paper, the windshield of a vehicle could be used for glasses-free 3D video calls. Of course, such a form of implementation could potentially increase the system requirements; however, the capabilities of small-scale variations (e.g., the system of Zhang et al. [144]) could be considered for this context.

## 6. Conclusions

In this paper, we presented our work on the visualization of V2X information in the context of automotive light field technology. We conclude that effective implementations of such a system must minimize the number of simultaneously visualized information in order to limit the cognitive load on the driver and that safety-related V2X warning messages should be prioritized. We also found that visualization should be context-aware so that the driver’s information deficit can be identified and properly addressed with the additional data. This identification process can correlate and, thus, simultaneously be executed with the V2X safety-related situation recognition of the vehicle’s V2X subsystem. Conveying the appropriate sensor data may increase the safety and efficiency of the traffic, and the well-defined viewing conditions of the utilization context enable resource-efficient system design. In future work, subjective studies with test participants should investigate the effect of such a solution on visual attention and cognitive load. Such analysis might cover various use cases in different driving conditions, such as platooning, RWW, or HLN. Furthermore, comparative studies should address the differences in information extraction efficacy between conventional displays (e.g., onboard units and smart devices) and light field visualization. Future research efforts should also aim to perform perceptual coding, which may result in the relaxation of system requirements while maintaining appropriate levels of perceived quality and user experience in general. As a long-term goal, novel studies and experiments may bring forth new international standards for such 3D automotive projection systems.

## Figures and Tables

**Table 1:** Considered V2X protocols for cooperative perception and information sharing.

Facilities Protocol	Title	Standard
CAM	Basic Set of Applications; Part 2: Specification of Cooperative Awareness Basic Service	ETSI EN 302 637-2 V1.4.1 (2019-04)
DENM	Basic Set of Applications; Part 3: Specifications of Decentralized Environmental Notification Basic Service	ETSI EN 302 637-3 V1.3.1 (2019-04)
SPATEM/MAPEM	Dedicated Short-Range Communications (DSRC) Message Set Dictionary J2735_201603/Basic Set of Applications; Part 3: Specifications of Decentralized Environmental Notification Basic Service	SAE J2735/ETSI TS 103 301 V2.1.1 (2021-03)
IVIM	Dictionary of in-vehicle information (IVI) data structures/Basic Set of Applications; Part 3: Specifications of Decentralized Environmental Notification Basic Service	CEN ISO/TS 19321:2020/ETSI TS 103 301 V2.1.1 (2021-03)
SSEM/SSREM	Dedicated Short-Range Communications (DSRC) Message Set Dictionary J2735_201603/Part 2: Applications and facilities layer common data dictionary	SAE J2735/ETSI TS 102 894-2 V2.1.1 (2022-11)
VAM	Dedicated Short-Range Communications (DSRC) Message Set Dictionary J2735_201603/Vulnerable Road User Safety Message Minimum Performance Requirements J2945/9_201703/Vulnerable Road Users (VRU) awareness; Part 3: Specification of VRU awareness basic service; Release 2	SAE J2735, SAE J2945/9_201703/ETSI TS 103 300-3 V2.1.2 (2021-04)
CPM	Basic Set of Applications; Analysis of the Collective Perception Service (CPS); Release 2/Intelligent Transport Systems (ITS); Cooperative Perception Services	ETSI TR 103 562 V2.1.1 (2019-12)/ETSI TS 103 324
SAEM	Facilities layer function; Part 1: Services Announcement (SA) specification	ETSI TS 102 890-1 V1.1.1 (2017-05)
PCM	Intelligent Transport Systems (ITS); Platooning; Pre-standardization study	ETSI TR 103 298 (2022-03)
MCM	Intelligent Transport Systems (ITS); Vehicular Communications; Informative report for the Maneuver Coordination Service	ETSI TR 103 578 (2022-11)

**Table 2:** Relevant ITS service groups and use cases.

ITS Service Group	Description	Example Use cases
In-vehicle signage (IVS)	The main task of IVS services is to inform road users (primarily vehicle drivers) about the actual permanent and dynamic road signs. Indications can be either advisory or mandatory.	– Dynamic Speed Limit Information – Dynamic Lane Management – Embedded Free Text – Shock Wave Damping – Parking Information
Hazardous Locations Notification (HLN)	HLN services essentially provide warning messages about potentially dangerous road sections. Road users approaching such road sections receive information about the location, type, and duration of the hazardous event if known.	– Accident Zone – Traffic Jam Ahead – Stationary/Slow Vehicle Warning – Weather Condition Warning – Temporarily Slippery Road – Animal or Person on the Road – Obstacle on the Road – Emergency Vehicle Approaching – Railway Level Crossing
Road Works Warning (RWW)	RWW ITS services aim to increase the safety of workers on or near the road and vehicles participating in traffic, reduce the number of accidents between vehicles and emergency/road operator vehicles, and comprehensively increase the comfort of road users.	– Lane Closure and other restrictions – Road Closure – Road Works Mobile – Winter Maintenance – Road Operator Vehicle in Intervention – Road Operator Vehicle Approaching
Signalized Intersections (SI)	SI services were created to provide safer, easier, and more efficient crossings with minimized environmental pollution at signalized intersections by sharing infrastructure information with road users and vehicle data with traffic light controllers.	– Signal Phase and Timing Information – Green Light Optimal Speed Advisory – Imminent Signal Violation Warning – Traffic Light Prioritization – Emergency Vehicle Priority
V2V-specific use cases	Services running on vehicles specified by the so-called Day 1 V2X system features and identified as the first set of use cases using V2V communications.	– Dangerous Situation Warning for Electronic – Emergency Break Light – Intersection Collision Warning – Motorcycle Approaching Information – Pre-crash sensing warning

**Table 3:** Summary and comparison of the scientific literature on immersive windshield interface technologies.

Research Effort	Year	Technology	Contribution
Wittmann et al. [134]	2006	Simulation	Review, proposal, evaluation
Charissis et al. [131]	2009	Simulation	Proposal, evaluation
Doshi et al. [135]	2009	AR	Proposal, prototype, evaluation
Wu et al. [136]	2009	AR	Proposal, prototype, evaluation
Prototype of Holografika ^1^	2013	Light field	Prototype
Bark et al. [132]	2014	AR	Proposal, evaluation
Gabbard et al. [130]	2014	AR	Review
Olaverri-Monreal et al. [71]	2014	Simulation	Proposal, evaluation
Rao et al. [133]	2014	AR	Review, proposal
Olaverri-Monreal et al. [72]	2016	AR	Review, proposal
Lee et al. [16]	2017	AR, light field	Review, proposal
Olaverri-Monreal et al. [137]	2017	Simulation	Proposal, evaluation
Sechrist [21]	2017	AR	Review
Kim et al. [73]	2018	AR	Proposal, prototype, evaluation
Olaverri-Monreal [138]	2018	Simulation	Review, proposal
Wang et al. [139]	2018	AR, light field	Proposal, prototype, evaluation
Doshi et al. [140]	2019	AR	Review
Bram-Larbi et al. [141]	2020	AR	Review, proposal
Lee et al. [17]	2020	AR, light field	Proposal, prototype, evaluation
Yöntem et al. [28]	2020	AR	Review, proposal, prototype
Wang et al. [74]	2020	Simulation	Proposal, evaluation
Deng et al. [18]	2021	AR	Proposal, prototype, evaluation
Lee et al. [19]	2021	AR, light field	Proposal, prototype
Maruta et al. [142]	2021	AR	Proposal, prototype, evaluation
Jeong et al. [20]	2022	AR	Proposal, prototype

^1^ Not published in the scientific literature.

## Data Availability

Not applicable.

## Abbreviations

The following abbreviations are used in this manuscript:

ABS	Anti-lock Braking System
ADAS	Advanced Driver Assistance System
API	Application Programming Interface
AR	Augmented Reality
AUV	Autonomous Underwater Vehicle
ASR	Anti-Slip Regulation
CA	Cooperative Awareness
CAM	Cooperative Awareness Message
CAV	Connected and Autonomous Vehicles
C-ITS	Cooperative Intelligent Transportation System
CP	Cooperative Perception
CPS	Cooperative Perception Service
DDS	Distributed Display System
DEN	Decentralized Environmental Notification
DENM	Decentralized Environmental Notification Message
DIS	Driver Information Systems
ECU	Electronic Control Unit
ESP	Electronic Stability Control
FOV	Field of View
FP	Full Parallax
GLOSA	Green Light Optimum Speed Advisory
GPS	Global Positioning System
HLN	Hazardous Locations Notification
HMI	Human-Machine Interface
HOP	Horizontal-Only Parallax
HUD	Head-up Display
I2V	Infrastructure-to-Vehicle
IMU	Inertial Measurement Unit
ISO	International Organization for Standardization
ITS	Intelligent Transportation System
IVIM	Infrastructure to Vehicle Information Message
IVS	In-Vehicle Signage
KPI	Key Peformance Indicator
LED	Light-Emitting Diode
MAC	Medium Access Control
MCM	Maneuver Coordination Message
OEM	Original Equipment Manufacturer
PCM	Platooning Control Message
RFID	Radio-Frequency Identification
RSU	Road-Side Unit
RWW	Road Works Warning
S3D	Stereoscopic 3D
SAE	System Architecture Evolution
SI	Signalized Intersections
SREM	Signal Request Extended Message
SSEM	Signal Request Status Extended Message
SPATEM	Signal Phase and Timing Extended Message
TLC	Traffic Light Control
V2I	Vehicle-to-Infrastructure
V2V	Vehicle-to-Vehicle
V2X	Vehicle-to-Everything
VAM	Vulnerable road user Awareness Message
VOP	Vertical-Only Parallax
VRU	Vulnerable Road User
VVA	Valid Viewing Area
